# Notes on the Medical Military Course at the Rockefeller Institute

**Published:** 1918-04

**Authors:** 


### Notes on the Medical Military Course at the Rockefeller Institute.

(Medical Officers are frequently ordered to institutions for special training. On their return to ordinary duty, they pass on the information by lectures and demonstrations, attendance on which is obligatory, though appreciated by most men as a privilege. The following are published by courtesy of the lecturer and with the approval of the Commandant of a Base Hospital).

BACILLUS AEROGENES CAPSULATUS. The natural habitat is the intestine of man, the dog and cat and other domestic animals and it is found in soil polluted with excreta, thus being especially prevalent in Belgium and northern France, along with the tetanus bacillus. During the first two years of the war, there was a high mortality from gas bacillus gangrene, especially after wounds from shrapnel, etc., which tend to blow fragments of soiled clothing or the soil itself into wounds. The bacillus grows very much like the Klebs-Loeffler, i.e., in a sense purely locally without invasion of the blood, but with extension along exposed surfaces, in the present instance especially fascial planes. In both diseases, the toxins are freely absorbed and produce profound depression. Locally, a marked oedema is produced, followed in a few hours by gangrene of the subcutaneous and muscular tissues which are ultimately reduced to a pulp. Efficient antiseptic treatment requires complete exposure by free incisions; for instance in a wound of the thigh, a long longitudinal and a transverse incision.

There strains of gas bacillus may be recognized, from each of which an antitoxin has been prepared after methods analogous to those for diphtheria, but it has been found that the antitoxin from any strain protects against all. Three times the lethal dose of toxin plus the antitoxin fails to cause death in guinea pigs, etc., and the practical therapeutic test is satisfactory. Owing to the universality of danger of gas bacillus and tetanus infection along the western front, a combined vaccine and antitoxin will be used.

The first human case treated with gas bacillus antitoxin was that of a boy of 16, who sustained a compound multiple fracture of the lower end of the left humerus and the olecranon. He had marked gangrene with the line of demarcation just above the condyles of the humerus. The temperature was over 105, the pulse 150-160 and the patient was unconscious. This was a case in civil practice. Dr. Welch injected the antitoxin subcutaneously and also in a ring above the line of demarcation—this latter procedure not being now considered necessary. In 7 hours, the temperature had fallen to less than 100 and the boy became conscious and demanded food. The next morning, amputation was done and recovery ensued.

The possibilities of anaphylaxis were brought out in the discussion. It was stated that, in the battle line, this would have to be disregarded. Anti-anaphylaxis could theoretically be secured by the use of a drop of the antitoxin every fifteen minutes for 4 to 5 injections but the delay would render this precaution out of the question in military surgery so that the slight risk of anaphylaxis must be incurred.

SHOCK. Whether due to haemorrhage or traumatism generally, or psychic, as in so-called shell-shock, an intraspinous injection of 3 c.c. of 1:1000 adrenalin solution is recommended. This will raise the blood pressure in a few minutes and will maintain it at normal from 1-1½ hours, when other measures may be employed. The solution is heated to drive off the chloretone used as a preservative, as otherwise depression might occur.

SUSPENDED RESPIRATION. A box, about 45 c.m. square, with a hinged bottom and slit rubber neck piece adjusted loosely, is placed over the patient's head. Carbon dioxide is introduced through a tube from a cylinder and respiration is stimulated by the temporary exclusion of oxygen. This method should probably not be used in cases directly due to asphyxiation.

FRACTURES. An effort is being made to standardize the treatment so far as possible. It is expected that a large percentage of the cases encountered will consist of compound comminuted fractures. Those shot in the abdomen and chest will figure to a less degree in the hospital statistics because such wounds will result fatally in most instances and especially unless the line is advancing rapidly in an attack so that the wounded can be picked up promptly.

The Thomas splint has, so to speak, been reborn. This consists in a curved rod to extend the length of the lower limb, with a ring at the top to pass around the thigh and to be supported largely from the ischium. It is used simply to immobilize; is to be applied at first dressing station; the patient is not to be removed from the original stretcher nor the splint removed till the base hospital is reached. Some modification of the Hodges splint, affording immobility of the fracture and relatively free mobility of the limb or body generally, will then be employed. Or the Balkin frame, perfected by several Americans, will be used.

CAREL-DAKIN TECHNIC. There has been a general misunderstanding that the authors of this method have insisted on its absolute perfection and that it is the sole method to be applied. They do not by any means declare that it is the last word in antiseptic treatment. What they do claim is that after Carel, Dakin and several French surgeons had experimented with over 200 different antiseptic solutions, this technic gave the best results in an average series of cases, especially when a routine treatment was employed without regard to special circumstances. The idea is the fundamental one of Lister, Pasteur and others, to render a septic wound aseptic by some substance freely soluble in the secretions, without introducing a generally toxic chemical nor one that will exert a depressing action on healthy tissues, and incidentally, the substance must not cause pain. The antiseptic action is a chemic process proceeding in the protein of the germs themselves. The practical test of the method is that in the second and third years of the war limbs were saved in 70% of the cases that required amputation in the first year. The solution is practically painless, odorless—a noticeable contrast to the experience in wards of similar cases under other methods—and a healthy granulating surface is rapidly achieved. The statement that wounds thus treated have the appearance of post-mortem specimens is not true.

On dressing the wound, smears are made from whatever sites still show small sloughs. 60 germs in a field are counted as infinity, an average of one to five fields as zero. Cocci and bacilli are distinguished, and all bacilli are considered as probably gas bacilli so that the infection would be rated as infinite, however small the count, so long as bacilli are present at all in five fields.

At the Rockefeller Institute, the nearest approach to war conditions, included compound fractures due to machinery, automobile accidents and the like, in which the wound was ground into street dirt. The wounds are freely incised and every particle of foreign matter and necrotic tissue picked out. Bone fragments are also removed, unless considered "orthopaedic," including both those with adherent periosteum and those necessary to serve as a wedge to hold other fragments in position, even though themselves devoid of periosteum. Foreign bodies and dirt must be sedulously removed from the medulla. Haemostasis must be as nearly perfect as possible, since neither Dakin's solution nor dichloramine T has any appreciable action on blood clot. When properly prepared, the wound resembles the clean surface of a piece of beef steak. The Dakin tubes are then introduced so as to reach every pocket and crevice, the number depending upon circumstances. A very small compress is applied and about 4 layers of gauze are laid on loosely before applying the retentive bandage.

As Dakin's solution produces a little dematitis in a few instances, the skin is covered with a single layer of thick-meshed, sterile, vaseline gauze. No drainage is used with Dakin's solution but it is used with dichloramine T.

The pad is made of two layers of half inch absorbent cotton and one inch of non-absorbent cotton outside, held in place with a little gauze bandage and the whole dressing is secured by small clip clothes pins such as may be obtained at 5 and 10 cent stores.

At ordinary room temperature, a sufficient quantity of Dakin's solution is introduced to wash out the secretion from a wound and this is repeated every two hours, it having been determined that the action is practically continuous for this length of time. The dressings are changed every day and the cultures are made every second day. Cotton is used for sponging, instead of gauze, to prevent irritation and, as it is generally recognized that it is extremely difficult to sterilize the skin absolutely, care is taken not to reinfect the wound from the skin in sponging.

The criticism that drainage is not provided is answered on the ground that necrotic material is removed in advance and that haemostasis is practically perfect, while chlorine is generated for a period of 1½ hours on the average. The use of the method in bed sores was considered advisable but with due regard for the fact that the myelitic element usually present would probably prevent the restoration of the trophic function.

---

**Chorea Cured by Spontaneous Abscess.**. Grenier de Cardenal, Jour. de Med. de Bordeaux, Oct. 1917, reports the case of a soldier aged 19, who developed polyarticular rheumatism in Jan., rejoined his command in March, and a few days later developed choreic movements of the maxilla and tongue, which soon became general. By May, he had reached a condition of profound malnutrition with the skin of almost the whole body covered with ecchymoses and excoriations and an abscess formed on the dorsum of the foot. It was opened and began to heal in a few days, the chorea disappearing and rapid recovery from the general lack of nutrition occurring.

**Citric Acid in Urine.** S. Amburg and W. B. McClure of Chicago, Am. Jour. of Physiology, Nov. 1917, have applied the Kunz method to the quantitation and find that at all ages, citric acid is eliminated in the urine, varying from 21 to 50 m.g. per 100 c.c. or, approximately 1/5 to 1/2 gram per day.

